# Efficacy of BRAF Inhibitors in Combination With Stereotactic Radiosurgery for the Treatment of Melanoma Brain Metastases: A Systematic Review and Meta-Analysis

**DOI:** 10.3389/fonc.2020.586029

**Published:** 2021-02-22

**Authors:** Muhammad Khan, Tao Zheng, Zhihong Zhao, Sumbal Arooj, Guixiang Liao

**Affiliations:** ^1^Department of Oncology, Shenzhen People’s Hospital, The First Affiliated Hospital of Southern University of Science and Technology, Shenzhen, China; ^2^Department of Oncology, First affiliated Hospital of Anhui Medical University, Hefei, China; ^3^Department of Nephrology, Shenzhen People’s Hospital, Second Clinical Medicine Centre, Jinan University, Shenzhen, China; ^4^Department of Biochemistry, University of Sialkot, Sialkot, Pakistan

**Keywords:** melanoma brain metastases, stereotactic radiosurgery, BRAF inhibitor, survival, treatment-related adverse events

## Abstract

**Background:**

BRAF inhibitors have improved the outcome for patients with BRAF mutant metastatic melanoma and have shown intracranial responses in melanoma brain metastases. Stereotactic radiosurgery (SRS) is being used as a local treatment for melanoma brain metastasis (MBM) with better local control and survival. We searched for studies comparing the combination of two treatments with SRS alone to detect any clinical evidence of synergism.

**Materials and Methods:**

PubMed, EMBASE, Medline, and Cochrane library were searched until May 2020 for studies with desired comparative outcomes. Outcomes of interest that were obtained for meta-analysis included survival as the primary, and local control as the secondary outcome.

**Results:**

A total of eight studies involving 976 patients with MBM were selected. Survival was significantly improved for patients receiving BRAF inhibitor plus SRS in comparison to SRS alone as assessed from the time of SRS induction (SRS survival: hazard ratio [HR] 0.67 [0.58–0.79], p <0.00001), from the time of brain metastasis diagnosis (BM survival: HR 0.65 [0.54, 0.78], p < 0.00001), or from the time of primary diagnosis (PD survival: HR 0.74 [0.57–0.95], p = 0.02). Dual therapy was also associated with improved local control, indicating an additive effect of the two treatments (HR 0.53 [0.31–0.93], p=0.03). Intracranial hemorrhage was higher in patients receiving BRAF inhibitors plus SRS than in those receiving SRS alone (OR, 3.16 [1.43–6.96], p = 0.004).

**Conclusions:**

BRAF inhibitors in conjunction with SRS as local treatment appear to be efficacious. Local brain control and survival improved in patients with MBM receiving dual therapy. Safety assessment would need to be elucidated further as the incidence of intracranial hemorrhage was increased.

## Introduction

Melanoma is a malignant, aggressive form of skin cancer associated with extensive disease and poor outcome (1, 2). It is the fifth leading cause of cancer, with an estimated 100,350 new cases, and 6,850 deaths expected to occur from melanoma in United States, in 2020. This characterizes a 6.8% mortality rate (3, 4). Therapeutic management of melanoma has undergone tremendous transformation with the United States Food and Drug Administration approving several new targeted and immunotherapeutic agents since 2007 (5, 6). Melanoma harbors mutually exclusive oncogenic mutations, including BRAF (50%), NRAS (20%), and KIT (1%) (6, 7). Targeted agents aimed at BRAF oncoprotein (vemurafenib; dabrafenib; encorafenib) and its downstream substrate, mitogen-activated protein kinase kinase (MEK, selumetinib; trametinib; cobimetinib; binimetinib), mainly affecting the mitogen-activated protein kinase (MAPK) pathway, have shown superiority over chemotherapy (8–12). The combination of BRAF and MEK inhibitors has further improved the outcome compared to single agents alone (13–16). In addition, immunotherapy targeting programmed cell death protein 1 and cytotoxic T-lymphocyte-associated protein 4 (CTLA-4) has also been added to improve outcomes in patients with metastatic melanoma (5). Subsequently, death rates have declined yearly by 7.0% in younger adults (<50 years old), and 5.7% in older patients, between 2013 and 2017 (3).

Melanoma is the third most common cancer type (10%), after lung (50%) and breast cancers (20%), that spreads to the brain. Furthermore, patients with melanoma are at the highest risk of developing brain metastases (10%–44%) (17, 18). Risk is increased to 75% in patients with metastatic melanoma (19). Autopsy series has revealed 80% central nervous system (CNS) involvement in patients with metastatic melanoma (19). Prognosis is poor for patients with melanoma brain metastases (MBM), and brain metastasis is the main contributor to mortality (up to 94.5%) in these patients (20). Management with surgery, chemotherapy, whole brain radiotherapy (WBRT), stereotactic radiosurgery (SRS), or their combinations has displayed a median survival of 3.8–7.69 months (19–21). MBM has been termed radioresistant. WBRT alone has been associated with limited local control and reduced median survival ranging from 2.86 to 3.86 months (19, 21, 22). SRS alone has shown better efficacy, with median survival times between 5.3 and 10.5 months, possibly due to better local control reported to be between 73% and 90% (23–31). The addition of WBRT to SRS has been inconclusive in this group of patients (29–31). As a result, a surge in SRS use was observed in patients with MBM from 2010 to 2015 due to radioresistance and late neurotoxicity associated with WBRT (32).

Outcomes for metastatic melanoma have improved impressively with targeted therapy and immunotherapy or their combinations (5–7). Studies have reported intracranial responses with targeted agents; however, their efficacy in melanoma patients with brain metastases has not been well established (33–36). Vemurafenib’s access to the brain was shown to be limited in preclinical studies involving ABCB1 and ABCG2 efflux pumps. Moreover, its hydrophobic and hydroscopic structure also suggests limited brain distribution (37–41). Nonetheless, vemurafenib has not only exhibited a protective effect against brain metastatic spread, but has also shown intracranial responses in several case reports, retrospective, and trial studies (33, 34, 42–47). Results of phase I/II trials have revealed that dabrafenib alone induced intracranial responses in melanoma patients with BRAF (V600E/G/L) mutations (35, 48). Dabrafenib and trametinib (MEK inhibitor) combinations have demonstrated intracranial activity in BRAF (V600E/D/K/R)-mutated MBM patients with or without local therapy induction (36, 49). Other BRAF inhibitor and MEK inhibitor combinations have also shown safety and intracranial activity, such as vemurafenib/cobimetinib, vemurafenib/trametinib, and encorafenib/binimetinib (50). Several studies have revealed the combination of SRS and BRAF/MEK inhibition to be safe and efficacious (51–54). However, whether the addition of BRAF/MEK inhibitors to SRS is synergistic and better than SRS alone has yet to be determined. Several retrospective studies have revealed conflicting outcomes regarding the synergistic efficacy of BRAF/MEK inhibitors plus SRS in the management of MBM (55–62). Here, we attempt to address this issue by systematically reviewing the literature and performing meta-analysis of the outcomes for a better clinical perspective.

## Materials and Methods

Guidelines were followed according to PRISMA (Preferred Reporting Items for Systematic Reviews and Meta-Analyses) (63). The protocol of this study is registered on PROSPERO: CRD42020185984.

### Inclusion Criteria

#### Patients and Study Types

Studies reporting comparative outcomes for the combination of BRAF inhibitors and SRS with SRS alone in the treatment of BRAF mutant melanoma brain metastases (MBM) were included.

#### Types of Interventions

The “experimental group” was administered BRAF inhibitors, namely vemurafenib (VMB) and dabrafenib (DAB), in combination with SRS, and the “control group” was managed with SRS alone.

#### Outcomes of Interest

The outcome of prime interest was overall survival (OS), whereas outcomes of secondary interest were brain control (local and distant control) and safety outcomes, including adverse events, intracranial hemorrhage, and radiation necrosis.

### Search Strategy

#### Databases

PubMed, EMBASE, Medline, and Cochrane library were searched until May 10, 2020. Various search terms relevant to the inclusion criteria were employed with language restricted to English. No restrictions were applied to the study design. Furthermore, relevant studies’ references were examined for additional studies.

#### Study Selection

Endnote X9 Software was used to import the studies obtained from the databases. The studies were then organized and screened for duplication. After removal of duplicates, further screening for title and abstract was carried out. Eligible studies were scrutinized with full text reading. Two independent reviewers finally selected eligible studies for inclusion. The third reviewer was consulted in case of any disagreements.

#### Data Extraction

Data extraction was carried out using the modified form of “The Cochrane Collaboration Data Collection form-RCTs and non-RCTs” Extracted data included studies’ attributes, design, first author, time period, publication year, number of participants, number of treated lesions, treatment regimens, and main efficacy and safety outcomes for the overall study group. Characteristics of the patients included age, sex, performance status (KPS), number of brain metastases, recursive partitioning analysis classes, diagnosis-specific graded prognostic assessment, and graded prognostic assessment class, if available. Furthermore, outcomes of interest (survival, brain local control, and safety) for treatment differences were extracted.

#### Assessment of Risk for Bias

Quality assessment was carried out using the modified checklist of Downs and Black aimed at assessing the methodological quality of non-randomized interventional studies (64). The checklist mainly covers four aspects of quality assessment: reporting, external validity, internal validity (bias and confounding), and statistical power. Twenty-seven questions are outlined, each carrying a score of one point, except for one question in the reporting section. Each section comprises of a different number of questions as follows: 10 questions in reporting, three questions in external validity, 13 questions in internal validity, and 1 question in statistical power. In this modified version, the statistical power question was also assigned a single point as opposed to the original, in which it carries five points. The modified version was used mainly for simplification and ambiguity avoidance (65). A grade was assigned according to the score obtained by each study as follows: excellent, if the score was between 24 and 28 points; good: 19–23 points; fair: 14–18 points, and poor: <14 points.

### Measurement of Treatment Effect and Data Synthesis

Hazard ratios for the treatment effect (survival and local control) were extracted directly from papers if given. When hazard ratios were not published, they were extracted from the Kaplan-Meier curves using the Digital Equalizer and methods for incorporating summary time-to-event data into meta-analysis according to Tierney et al. (66). A similar approach was also applied for local control data. The acquired hazard ratios were pooled using the software “RevMan 5.3 software” (67, 68). An inverse variance statistical method was applied for pooling hazard ratios using the fixed effects analysis model. The significance level (P value) was set at <0.05. Heterogeneity was assessed using Chi^2^ test and I^2^ value. I^2^ values of 25%, 50%, and 75% were considered low, moderate, and high, respectively (69). A random effects analysis model was used in case of moderate heterogeneity (50%).

## Results

**Figure 1:**
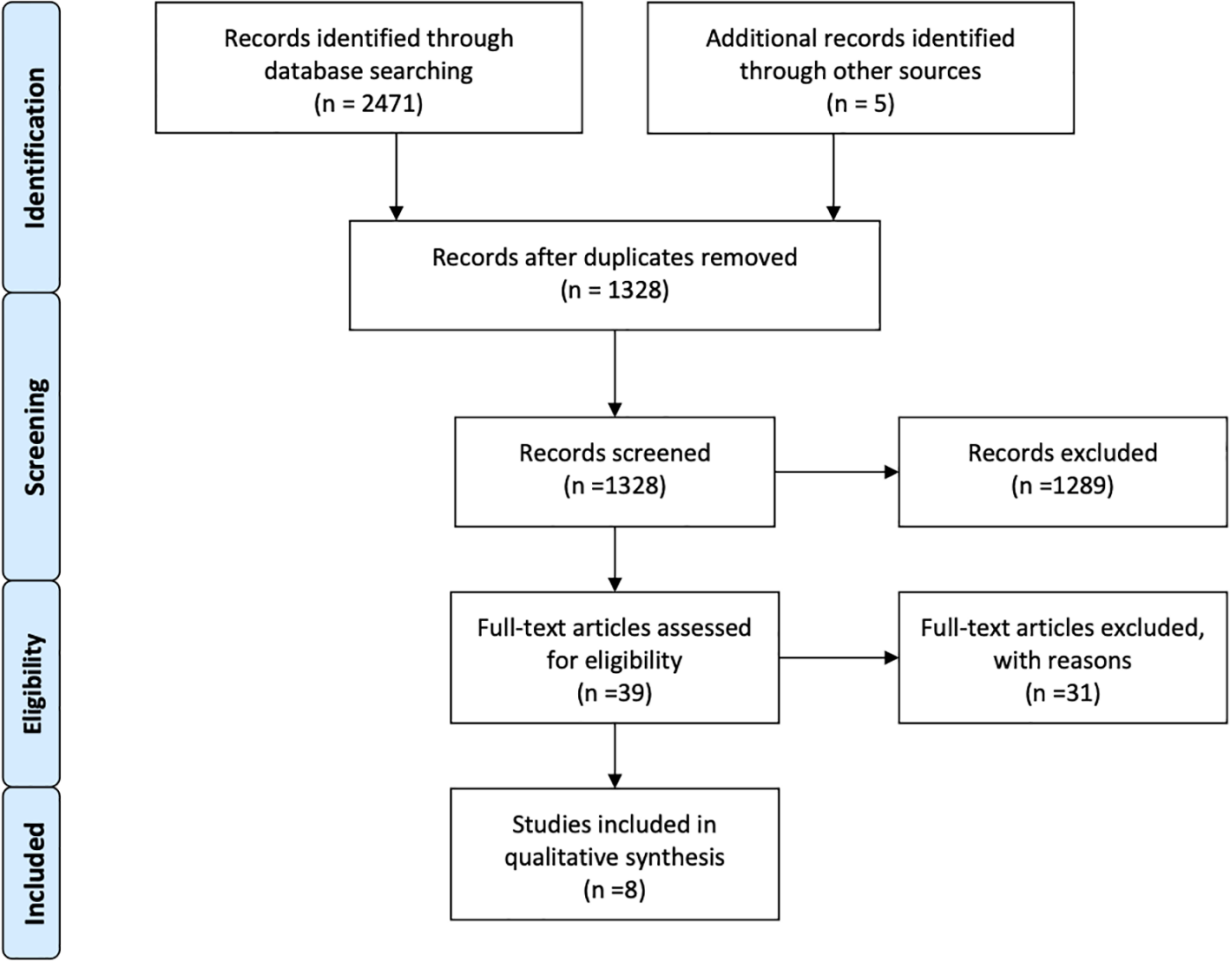
Flow diagram of research strategy and study selection.

Overall, eight retrospective studies, that met the inclusion criteria, were identified after a comprehensive research and selection process (**Figure 1**) (55–62). A total of 976 MBM patients had either received SRS alone (n = 728) or BRAF inhibitors plus SRS (n = 244) for management of their brain disease. Majorly, vemurafenib and minorly dabrafenib were used as the main choice of BRAF inhibitor. One study also used an MEK inhibitor in addition to a BRAF inhibitor (59). SRS was used as the main local brain radiation therapy, except in one study in which upfront WBRT and surgery were also applied. However, only survival outcomes were obtained regarding these participants. Other outcomes, such as local control, distant control, and side effects were obtained from patients only in the SRS recipients’ subgroup (61). Two studies also included cohorts receiving immunotherapy; hence, specific comparative baseline characteristics were not available (58, 59). The general characteristics of the studies are reported in **Table 1**.

**Table 1:** General features of included studies and baseline characteristics of patients.

Studies	Period	Number	lesions	Age (median [range]) (yrs)	Gender (male/female)	Radiation	BRAF inhibitor	BRAF mutation	Median OS	LC	DC	AREs	MedianFollow-up	QA
***Ly et al*. (**55**)**	2009–2012	52	198	52 (19–64)	40/12	SRS	VMF and DAB	31	12 months	LC:69.2%	DC: 33.0% MTTDF: 5.5 months	ICH Before SRS: 27(13.6%) After SRS: 50 (29.2%)	10.5 months	20
***Patel et al*. (**56**)**	2005–2013	87	157	56 (35–65)	66/21	SRS	VMF and DAB	NA	NR	LF: 17%, MTTLR: 4.37 [0–18] months	DF: 71.3% (62)	NR	6.5 [0.4–152.3] months	17
***Wolf, et al*. (**57**)**	2012–2015	80	410	59.8 ± 14.6	50/30	SRS	VMF and DAB	35	BM: 9.7 [6.7–12.6] SRS: 6.7 [5.3–8.1] months	LC: 92.5% ± 22.3% MTTICP/NM: 2.2 [range, 0.4–16.6] months		ICH: 13.8%		20
***Gaudy-Marqueste et al*. (58)**		63				SRS	VMF and DAB	63	NA	NR	NR	NR		16
***Choong et al*. ** (59)	2010–2015	65				SRS	BRAF/MEKi	51	14.2 [8.8–20.4] months	BC at -6 m: 67% -12 m: 48%		RN: 1	8.6 [0.4–39.6] months	17
***Xu et al*. ** (60)	2010-2014	65	436	61.0 ± 12.7	39/14	SRS	VMF and DAB	30	MST: PD: 46 BM: 9 SRS: 6 months	LC: 89.4%		ARE: 20 ICH: 9 RN: 6		20
***Kotecha et al*. ** (61)	1987–2014	366 -SRS subgroup: 191	1336 -SRS subgroup: 793	57.3 ± 14.0	227/139	SRS WBRT Surgery	BRAFi	35 -SRS subgroup: 19	MST: 6 months OSR at -6 m: 50%, -12 m: 24%, -18 m: 17%	LF: 116 (32%) SRS subgroup:118 (15%)	DF: 144 (39%) SRS subgroup: -6 m: 48% -12 m:69% (-12 m-CI)	RN: 45 (12.2%)	SRS subgroup: 7 [range 1–174] months	21
***Mastorakos et al*. ** (62)	2011–2015	198	710	55.3 ± 15.3	134/64	SRS	VMF and DAB	90	MST: PD: 61 BM: 10.9 SRS: 8.1 months			ICH: 5.5%		21

SRS, stereotactic radiosurgery; VMF, vemurafenib; DAB, dabrafenib; OS, overall survival; BC, brain control; LC, local control; DC, distant control; LF, local failure; MTTLR, median time to local recurrence; BM, survival from brain metastases; SRS, survival from SRS initiation; WBRT, whole-brain radiation therapy; AREs, adverse radiation events/effects; RN, radiation necrosis; QA, quality assessment. MTTICP/NM, median time to intracranial progression/new metastases; PD, survival from primary diagnosis; CI, cumulative incidence.

### Baseline Characteristics of Patients

There were some significant differences in patient characteristics between the two groups. BRAF mutant patients were younger than the patients with wild-type BRAF. These differences were reported in four studies (55, 57, 61, 61). We used the data from four studies to perform meta-analysis of the age differences for BRAF mutant versus BRAF wild type as well as for BRAF inhibitor users versus non-users (57, 60, 61). Patients in the “BRAF mutant” and “BRAF inhibitor users” cohorts were comparatively younger (**Figures S1**, **S4****)** (**Table 2**). Mastorakose et al. (62), as well as Kotecha et al. (61) reported that patients with BRAF mutations were diagnosed with primary melanoma at a relatively younger age, 49 vs. 61 years and 60 vs. 64 year, respectively. This difference was maintained at the consequent BM diagnosis (58 vs. 66) (p<0.01) (61). Therefore, it could be speculated that BRAF mutations may expedite the process of oncological onset.

**Table 2:** Age and gender prevalence according to BRAF mutation and BRAFi therapy status.

Characteristics	No. of studies	No of Participants	Mean Difference/Odds ratio	Heterogeneity (I^2^)	Analysis Method
***BRAF mutant vs. BRAF w-t***					
***Age***	4	421	-9.58 [-13.72, -5.43], p<0.00001	58%	IV, Random
***Male***	6	560	0.52 [0.36, 0.75], p=0.0005	32%	M-H, Fixed
***Female***	6	560	1.94 [1.34, 2.81], p=0.0005	32%	M-H, Fixed
***BRAFi vs. Non-BRAFi***					
***Age***	6	793	-6.82 [-13.08, -0.56], p=0.03	82%	IV, Random
***Male***	8	932	0.76 [0.40, 1.47], p=0.42	62%	M-H, Random
***Female***	8	932	1.84 [1.30, 2.60], p=0.0005	5%	M-H, Fixed

Similarly, male sex was also identified as the predominant sex in the BRAF wild-type cohorts in two studies (57, 62). We performed a meta-analysis of six studies and the result revealed a significant predominance of male sex in BRAF wild type, but not amongst BRAF inhibitor non-users (**Figures S2**, **S5**) (55–57, 60–62). Female sex was more predominant amongst BRAF mutant and BRAF inhibitor receivers (**Figures S3**, **S6**) (55–57, 60–62). As for previous therapies, in the study by Patel et al., patients in the SRS alone group received significantly more systemic chemotherapy. In addition, studies have allowed for inclusion of patients with previous therapies before SRS for BM. However, an open label, single arm, phase 2 trial of vemurafenib found no differences between the cohorts that were separated by the status of previous therapy for intracranial responses, progression-free survival, and median survival (34). Similar results were also revealed for dabrafenib in a separate phase 2 trial (35). Kotecha et al. had allowed patients to receive upfront surgery, SRS, and WBRT. SRS was predominantly administered to patients receiving BRAF inhibitors. However, only survival outcomes were observed in this population. Other outcomes, such as local control and distant brain control, were extracted from the subgroup analysis of SRS recipients (n=119). BRAF inhibitor-receiving patients had better KPS scores than those receiving SRS alone (61). No significant differences in the patients’ baseline characteristics between the groups were reported other than those mentioned above. The baseline characteristics and main outcomes of the studies are outlined in **Table 1**.

### Overall Survival

Survival outcome for treatment comparison was reported in eight studies involving 976 MBM patients (55–62). Seven studies were used to synthesize the survival meta-outcome involving 924 patients (56–62). Survival outcome was analyzed in three categories: survival from the time of SRS induction, BM diagnosis, and primary diagnosis.

#### SRS Survival

**Figure 2:**
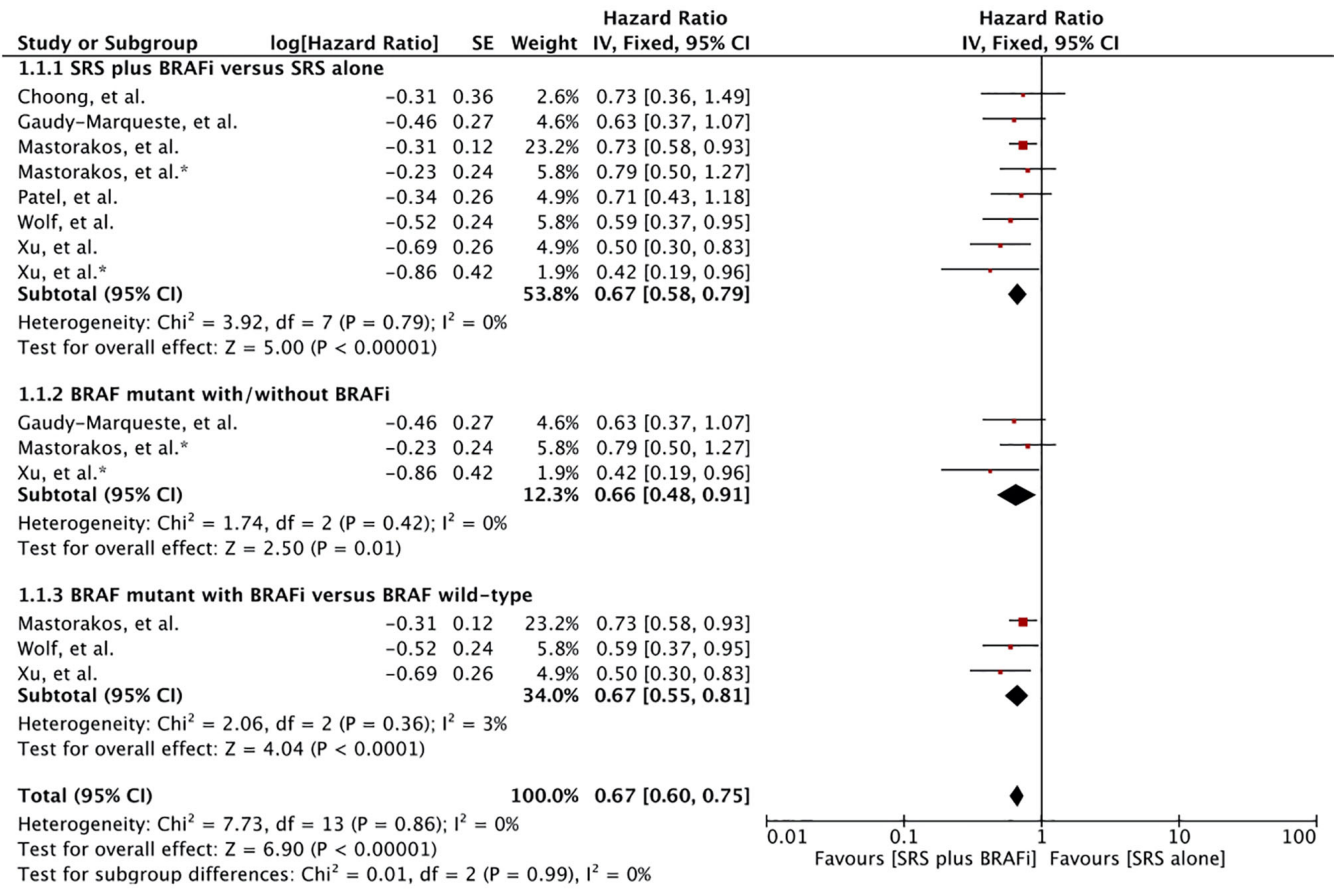
Forest plot of meta-analysis of overall survival (OS) from the time of stereotactic radiosurgery (SRS) induction (SRS survival) for treatment comparison (BRAF inhibitors plus SRS versus SRS alone) in the management of melanoma brain metastases (MBM). Subgroup analysis included comparison of BRAF-mutant patients receiving BRAF inhibitors and BRAF-mutant without BRAF inhibitors (Subgroups 1.2.2), and patients with BRAF-mutant receiving BRAF inhibitors and BRAF wild-type (Subgroup 1.2.3) for treatment comparison. *Represents comparison of BRAF mutant patients with/without BRAF inhibitor therapy.

Seven studies comprising 610 patients reported survival from the time of SRS induction (55–60, 62). Meta-analysis of six studies with eight survival outcome comparisons for treatment difference showed a significant advantage among the receivers of BRAF inhibitors in combination with SRS (56–60, 62). A significant hazard ratio of 0.67 with 95% confidence interval from 0.58 to 0.79 was revealed (p < 0.00001). No heterogeneity was observed among the studies (I^2^ = 0) (**Figure 2**). One study with 52 MBM patients reported no survival difference between the BRAF inhibitor cohort (n = 17) and non-BRAF inhibitor cohort (n = 14) (p = 0.82). Overall survival did not change when the patients with wild-type *BRAF* were included in the analysis (n = 35) (p = 0.90) (55).

Subgroup analysis was performed to compare MBM patients receiving BRAF inhibitors with BRAF-mutant patients not receiving BRAF inhibitor and BRAF wild-type alone (57, 58, 60, 62). Patients receiving BRAF inhibitors in addition to SRS were at a significant advantage in each comparison: BRAF mutant (HR 0.66 [0.48, 0.91], p = 0.01), and BRAF wild type (HR 0.66 [0.55, 0.81], p < 0.00001) (**Figure 2**).

#### BM Diagnosis Survival

**Figure 3:**
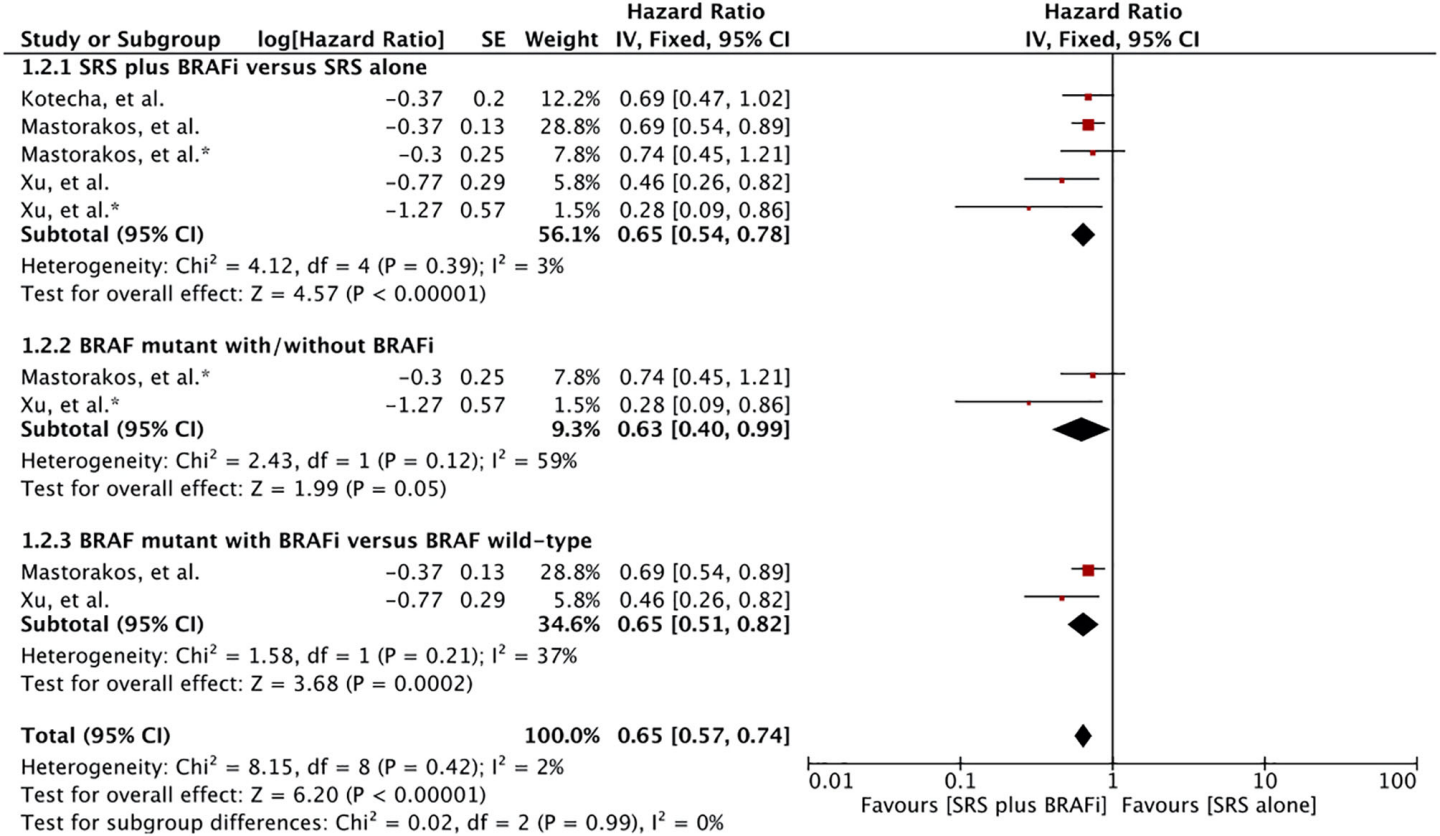
Forest plot of meta-analysis of overall survival (OS) from diagnosis of brain metastases (BM survival) for treatment comparison (BRAF inhibitors plus stereotactic radiosurgery [SRS] versus SRS alone) in the management of melanoma brain metastases (MBM). Subgroup analysis included comparison of BRAF-mutant patients receiving BRAF inhibitors and BRAF mutant without BRAF inhibitors (Subgroups 1.3.2), and patients with BRAF mutant receiving BRAF inhibitors with BRAF wild-type (Subgroup 1.3.3) for treatment comparison. *Represents comparison of BRAF mutant patients with/without BRAF inhibitor therapy.

Four studies involving 629 MBM patients reported post-BM diagnosis survival (57, 60–62). In addition to the comparisons previously described, one study also involved MBM patients with unknown BRAF mutation status. Meta-analysis of these three studies with five comparison outcomes revealed a survival advantage for MBM patients receiving BRAF inhibitor-SRS combination (HR 0.65 [0.54, 0.79], p <0.00001) (**Figure 3**) (60–62). In addition, Wolf et al. also reported significantly better survival in MBM patients with BRAF inhibitors than in BRAF wild-type patients (57). A median survival time of 13.2 months (95% CI: 8.1–8.3) versus 6.9 months (95% CI: 4.4–9.3) was revealed for treatment difference (p = 0.04).

Subgroup analysis revealed survival advantage for patients receiving BRAF inhibitors plus SRS in comparison to the BRAF mutant without BRAF inhibitor (HR 0.63 [0.40, 0.99], p=0.05) (**Figure 3**). However, the heterogeneity was more than 58%. After applying the random effects model, treatment comparison showed no significant difference (HR 0.52 [0.21, 1.30], p=0.16) (**Figure S7**). Patients receiving dual therapy had a significant advantage than wild-type BRAF (HR 0.65 [0.51, 0.82], p = 0.0003) (**Figure 3**). Subgroup analysis was based on results from only two studies (60, 62).

#### Primary Diagnosis Survival

**Figure 4:**
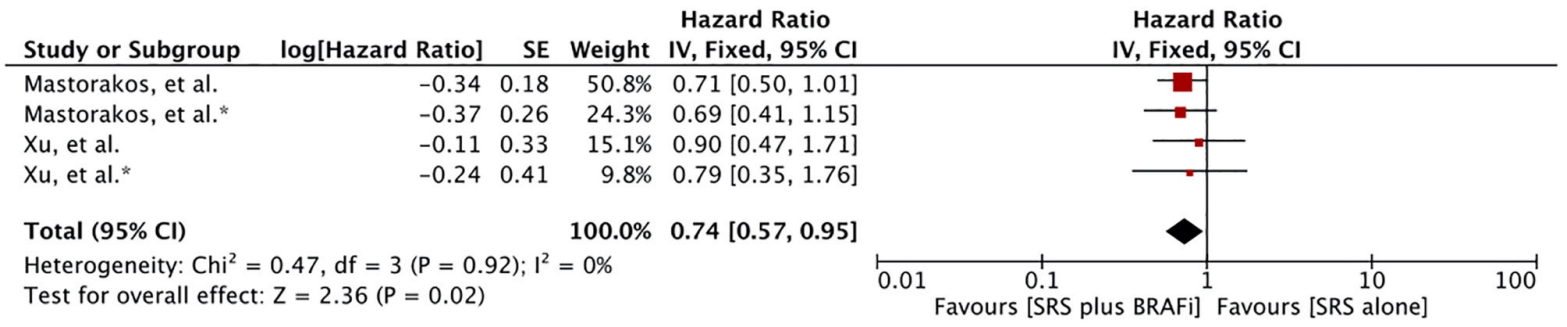
Forest plot of meta-analysis of overall survival (OS) from diagnosis of primary melanoma (PD survival) for treatment comparison (BRAF inhibitors plus stereotactic radiosurgery [SRS] versus SRS alone) in the management of melanoma brain metastases (MBM).

Meta-analysis of the survival difference between the two comparative treatments from the time of primary melanoma diagnosis revealed a significant overall survival advantage for BRAF inhibitor receivers (HR 0.74 [0.57, 0.95], p=0.02) (**Figure 4**). The meta-analysis was based on the results of two studies providing four comparative outcomes (60, 62). However, Wolf et al. revealed no difference in survival from the time of diagnosis of primary melanoma (MST 68.7m vs. 56.0m, p = 0.10) (57).

### Timing of BRAF Inhibitors’ Initiation

**Figure 5:**
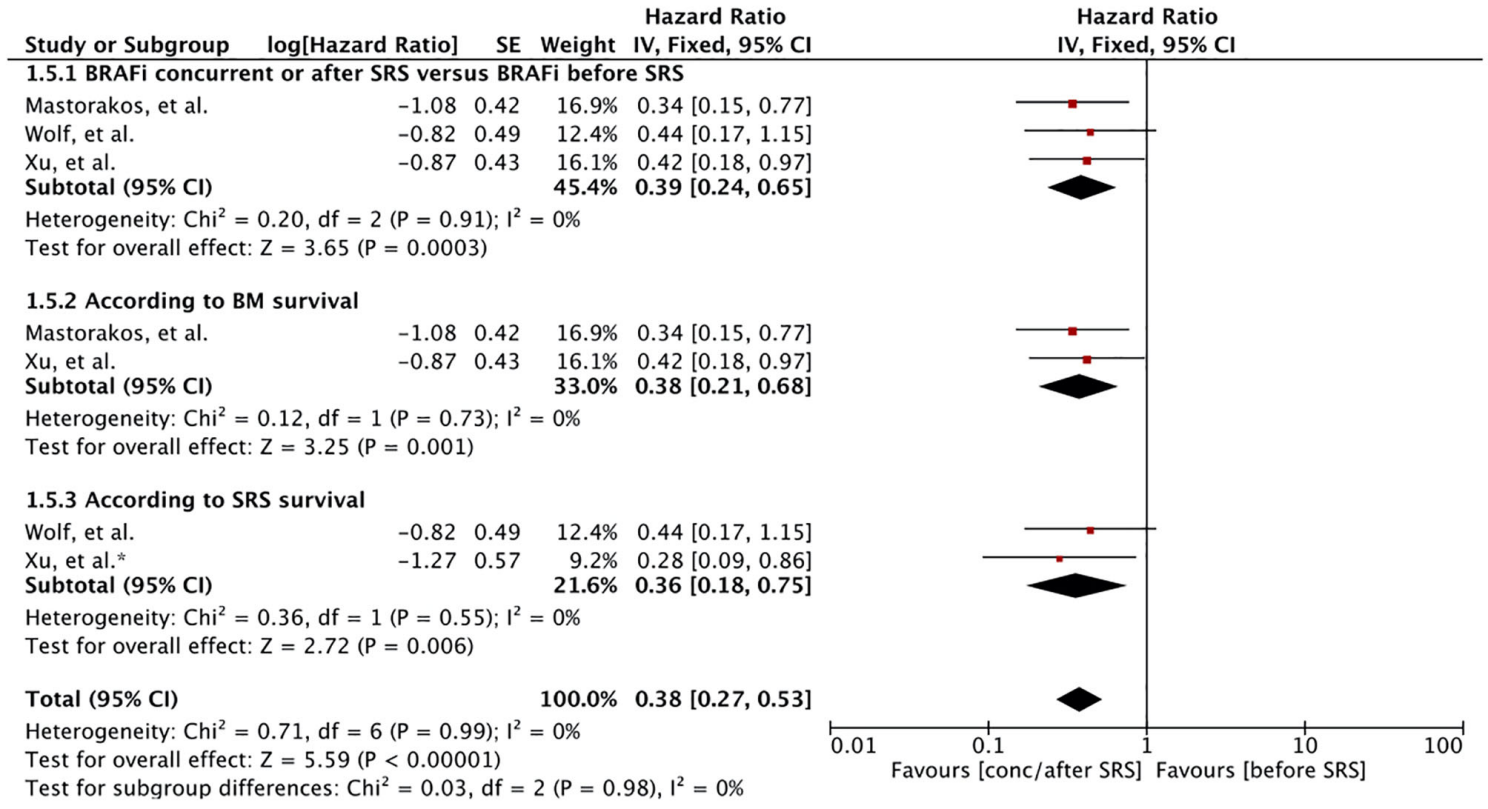
Forest plot of meta-analysis of overall survival (OS) for treatment comparison according to timing of BRAF inhibitors induction (BRAF inhibitors concurrently or after stereotactic radiosurgery [SRS] versus BRAF inhibitors before SRS) in the management of melanoma brain metastases (MBM). Subgroup analysis included comparison of treatment according to BM survival (subgroup 1.5.2) and SRS survival (subgroup 1.5.2).

Three studies involving 128 MBM patients reported survival based on BRAF initiation to SRS or BM development (57, 60, 62). Majority of the patients had received BRAF inhibitors after SRS. The patients with BRAF mutant not receiving BRAF inhibitors in the study by Xu, et al. had received BRAF inhibitor before BM development (n=10) (60). Meta-analysis of patients receiving BRAF inhibitors concurrently or after SRS induction showed that there had an advantage compared to patients receiving it before SRS (HR 0.39 [0.24, 0.65], p = 0.0003) (**Figure 5**). Survival after BM diagnosis remained significant for patients receiving BRAF inhibitor after SRS when restricting the results to studies assessing survival after BM diagnosis (HR 0.38 [0.21, 0.68], p = 0.001) (**Figure 5**). A similar result was obtained when survival after SRS was considered (HR 0.36 [0.18, 0.75], p = 0.006) (**Figure 5**). Mastorakose et al. also evaluated the survival difference between patients receiving BRAF inhibitors concurrently or after (62). Patients receiving BRAF inhibitor after SRS were found to have better survival (24 months vs. 10.1 months, p = 0.007).

### Local Control

**Figure 6:**
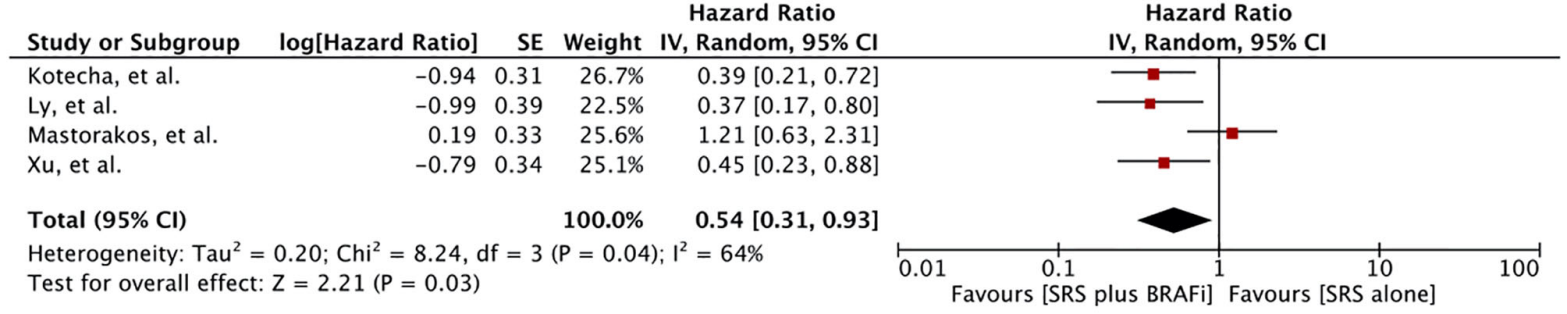
Forest plot of meta-analysis of local control (LC) for treatment comparison [BRAF inhibitors plus stereotactic radiosurgery (SRS) vs. SRS alone] in the management of melanoma brain metastases (MBM).

Local control rate was reported in six studies involving 395 patients (55–57, 60–62). Meta-analysis of four studies revealed a significantly better local brain control for patients receiving BRAF inhibitors in addition to SRS (HR 0.54 [0.31, 0.93], p = 0.03) (**Figure 6**) (55, 60–62). Heterogeneity was observed (I ^2^ = 64%). Excluding the study by Mastorakose et al., heterogeneity was reduced to 0%, although a significant difference was still maintained (HR 0.40 [0.27, 0.60], p<0.00001) (62). Two other studies also evaluated local control (56, 57). Patel et al. reported local failure in 15 (17%) patients with a median time to local failure of 4.37 months (0–18 months). No difference in the recurrence rate was observed in his study between the two groups (3.3% vs. 9.6% at 1 year, p = 0.43) (56). Wolf et al. also reported no difference in overall local control (94.6% ± 20.8% vs. 90.8% ± 25.2%, p=0.51). However, in their study, time to progression or new metastases was significantly longer for the BRAF inhibitor group (Median time: 3.9 months, range 0.8–16.6 months) than for the BRAF-*wt* group receiving SRS alone (Median time: 1.7 months, 0.4–9.3 months) (p = 0.02) (57).

### Distant Failure

Four studies reported distant intracranial failure involving 528 patients (55, 56, 61, 62). Ly et al. revealed no difference in distant brain failure in BRAF mutant patients between those who received BRAF inhibitors (n = 17) and those who did not (n = 14) (p = 0.97) (55). BRAF-mutant patients receiving BRAF inhibitor also showed no difference in distant brain failure when compared to a combined cohort of BRAF-mutant and BRAF wild-type patients not receiving BRAF inhibitors (n = 35) (p = 0.54). Another study with similar cohorts involving 198 patients also reported no difference in remote failure rates between BRAF-mutant with BRAF inhibitors (n = 67), BRAF-mutant with no BRAF inhibitor (n=23), and BRAF wild-type without BRAF inhibitor (n = 108) (p = 0.183) (62). Patel et al. also revealed no statistical difference in distant intracranial failure rates between SRS alone (n=72) and SRS plus BRAF inhibitor (n=15) groups (56). At 6-month and 1-year, distant intracranial failure rates (DIF) were 35.0% and 63.9% in the SRS alone group compared to 53.2% and 65.1% in the SRS plus BRAF inhibitor group, respectively (p = 0.45). One study (n = 191), in which BRAF inhibitors were used within 30 days of SRS in a small cohort (n = 19, lesions = 81) demonstrated a significant reduction in 12-month cumulative incidence of distant failure compared to patients with SRS alone (68% vs. 95%, p = 0.03) (61).

### Progression Free Survival

Only one study assessed brain progression-free survival for the treatment difference, revealing longer brain progression-free survival (BPFS) for patients receiving a combination of the two treatments (p = 0.042) (62).

### Safety Profile

The safety of the BRAF inhibitor-SRS combination was evaluated in several studies using various factors. These included, the rate of adverse radiation effects, adverse events, intracranial hemorrhage, and symptomatic and asymptomatic radiation necrosis.

#### Adverse Events

Several adverse events have been reported with BRAF inhibitors. Two studies reported the adverse events caused by vemurafenib and dabrafenib separately (60, 62). We have outlined the events in **Table 3**. Various adverse events, such as elevation of alanine transaminase, arthralgia, squamous cell carcinoma of the skin, seizures, and hyperkeratotic reactions were only associated with vemurafenib. Overall, dabrafenib was shown to have caused fewer adverse events compared to vemurafenib (18/30 versus 51/48). Three patients had to discontinue vemurafenib due to severe skin rash in the study by Xu et al. (60).

**Table 3:** Adverse radiation events in patients treated with BRAFi after the development of BMs.

Variables	Dabrafenib	Vemurafenib	Both	Total	Percentage (%)
***No. of patients***	30	48	6	84	
***Elevation of alanine transaminase***		4	1	5	6.0
***Arthralgia***		7		7	8.3
***Squamous cell carcinoma of the skin***		2		2	2.4
***Seizure***		1		1	1.2
***Rash***	4	10	1	15	17.9
***Myalgia***	1	4		5	6.0
***Diminished appetite***	5	3		8	9.5
***Hyperkeratotic reaction***		6		6	7.1
***Fatigue***	2	6		8	9.5
***Abdominal pain***	2	2		4	4.8
***Intracranial hemorrhage***	4	6	1	11	13.1
***Total events***	18	51	3	72	

#### Intracranial Hemorrhage

**Figure 7:**
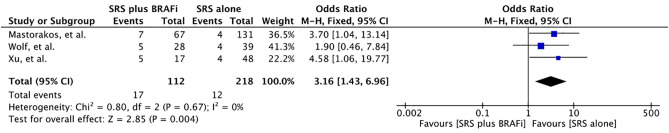
Forest plot of meta-analysis of intracranial hemorrhage (ICH) for treatment comparison [BRAF inhibitors plus stereotactic radiosurgery (SRS) vs. SRS alone] in the management of melanoma brain metastases (MBM).

Three studies involving 330 patients reported the number of patients developing intracranial hemorrhage (ICH) following BRAF inhibitor therapy (57, 60, 62). Meta-analysis of these studies revealed a significant increase in the odds for patients receiving both agents compared to SRS alone (OR 3.16 [1.43, 6.96], p=0.004) (**Figure 7**). However, Patel et al. reported that ICH rates at lesion level showed no increase in patients taking BRAF inhibitors [SRS: 12%/(125) vs. SRS+BRAF inhibitor: 34.4%/(32)] (56). One study also evaluated the freedom from intracranial hemorrhage between the groups. The 1-year freedom from ICH rate was 39.3% for patients receiving BRAF inhibitors compared to 77.0% for patients without BRAF inhibitor (p=0.0003) (55).

#### Radiation Necrosis

Considering the number of patients who developed radiation necrosis (RN), there was no difference between the treatments based on two studies (56, 60). Both Xu et al. (60) and Patel et al. (56) assessed the difference based on the number of patients and found no significant difference in the number of RN at lesion level between the two groups [SRS: 3.2% (125) vs. SRS+BRAF inhibitor: 0% (32)] (56). However, using the cumulative incidence model statistics, a significant increase in the RN (22.2% vs. 11.0%, p<0.001) and SRN (28.2% vs. 11.1%, p<0.001) at 1 year for patients receiving BRAF inhibitors delivered in proximity to SRS. On the other hand, Kotecha et al. revealed a lower 12-month cumulative incidence of RN for lesions treated with BRAF inhibitor (0% vs. 6%, p=0.04) (61).

### Publication Bias

Publication bias was assessed using a funnel plot for overall survival. All results were within the 95% CI indicating no evidence of publication bias in the SRS, BM, and primary diagnosis (PD) survival outcomes (**Figures S8**–**S10**).

## Discussion

Brain metastases are common in metastatic melanoma and are associated with poor prognosis (18–21). Approximately 20% of patients with metastatic melanoma have brain metastasis at the time of diagnosis; over 50% develop these at some point during the course of the disease (18, 19, 70). Management of MBM includes surgery, SRS, WBRT, and cytotoxic chemotherapy (18). The addition of targeted agents and immunotherapy has improved the outcome significantly (5–7). SRS has been increasingly used as the treatment of choice for local therapy (32). In fact, the combination of radiation therapy and immune checkpoint blockers, such as ipilimumab and nivolumab has shown synergistic responses in various retrospective studies (53, 58, 59, 71–74). BRAF inhibitors have also shown intracranial responses, suggesting that the two treatments could work synergistically (33–36). Preclinical evidence suggests that the MAPK pathway, the pathway targeted by BRAF or MEK inhibitors, is activated following ionizing radiation, resulting in cell proliferation, differentiation, and survival. *In vivo* and *in vitro* inhibition of the MAPK signaling pathway was able to reverse these ionizing radiation effects (75, 76). Ex vivo analysis of chromosomal breaks in patients treated with radiation plus BRAF inhibition showed increased radiosensitivity in patients treated with vemurafenib (P = 0.004) and vemurafenib switched to dabrafenib (P = 0.002). Dabrafenib was not shown to increase radiosensitivity in this study (77). The occurrence of skin toxicity (dermatitis) on previously irradiated skin in patients receiving vemurafenib was also suggestive of vemurafenib being a radiosensitizer (78). Thus, preclinical and clinical evidence suggests that combining the two treatments could lead to synergistic responses, thereby improving the survival outcome.

Based on the evidence from eight studies, our results indicate that patients receiving SRS plus BRAF inhibitors had significantly better survival benefits. In a retrospective study, patients receiving BRAF inhibitors along with SRS also showed a similar surge in survival for MBM patients (18 months vs. 5 months, *p* = 0.009) (79). However, the patients had also used anti-CTLA-4 monoclonal antibodies, and the proportion of each drug was not specified, for which this study was excluded from our analysis. Only one study failed to report any survival advantage (55). The ICH rates for BRAF mutant and BRAF wild-type before treatment were compared (19/127 vs. 8/50, p=0.86). However, in a comparison of BRAF-mutant with BRAF inhibitors and non-BRAF inhibitor users, the rate was not specified. Evidently, hemorrhagic MBMs are associated with lower local control after SRS, and may lead to decreased survival for such patients (80). Fifteen of the 20 deaths attributed to CNS etiology were from ICH, indicating the ICH impact on survival analysis (55). In subgroup analysis, we observed a trend for patients with BRAF mutations receiving BRAF inhibitors to achieve far better survival than BRAF wild type and BRAF mutant without BRAF inhibitor use. The trend was maintained regardless of whether the survival assessment was from SRS or BM diagnosis. This is in contrast to studies associating worst survival with BRAF mutation status before the era of BRAF inhibitors (32, 81, 82). One reason could be the small number of patients in the comparative groups for BRAF mutants with/without BRAF inhibitors. In addition, current studies have been undertaken during the era of immune checkpoint inhibitors as immunotherapy, and these operate synergistically with SRS (53, 58, 59, 71–74). Therefore, patients without BRAF inhibitors may have opted for such therapies, thereby improving the outcome (56, 60, 62). It has also been pointed out that BRAF mutation was associated with improved local control in patients with MBM, which may imply higher radiosensitivity, thereby eliciting better response than patients with BRAF wild-type (83). On the other hand, our finding is consistent with that of Menzies et al., who also revealed significant differences in 1-year OS rates for patients with BRAF-mutant BRAF inhibitor use (83%) compared to BRAF wild-type (37%), and BRAF mutant without BRAF inhibitor use (29%) (p<0.001) (84). Another important observation was the effect of induction timing of BRAF inhibitors with respect to SRS or BM development. Patients receiving BRAF inhibitors concurrently or after SRS were shown to have superior survival than patients receiving it before SRS. Similar observations were made in studies involving patients with renal cell carcinoma (RCC) and BM. In this study, patients receiving tyrosine kinase inhibitors (TKIs) after BM development had a significantly better survival advantage compared to patients developing BM while they were on TKIs (23.6 months vs. 2.08 months, p=0.0001) (85). This could reflect the higher sensitivity of patients to BRAF inhibitors receiving it for the first time. Added advantage could also come from the systemic disease control of these patients as well as better brain control.

Improved local control was also revealed based on data from four studies. Improvement in local control demonstrates that the survival benefit may be a result of synergism between the two treatments. Even though BRAF inhibitors have been shown to have limited brain penetration, the fact that SRS may focally disrupt the blood brain barrier by targeting the vasculature could possibly pave the way for targeted agents to reach the tumor (86, 87). It is also hypothesized that targeting driver mutations with high specificity may lower the concentrations required for radiosensitization (83). Most of the studies revealed no difference in distant failure between the treatments. It could be hypothesized that SRS could only disrupt the blood brain barrier locally, thus leaving the distant control unaffected (86, 87). Furthermore, acquired resistance to BRAF inhibitors could also lead to distant failure (88). Moreover, a significant delay in distant failure reported in one study may be a manifestation of a better response from the fact that BRAF inhibitors used after SRS were reported to have improved survival outcomes compared to concurrent use or use before SRS (57, 61, 62).

From a safety perspective, BRAF inhibitors have been associated with skin toxicity, ICH, and RN (33–36, 55, 56). Only one study elaborated the adverse events excluding RN in a comparative manner (60). No difference was revealed in that study between the treatments. Instead, a slower rate of adverse radiation effects was observed in patients receiving BRAF inhibitors. Intracranial hemorrhage was significantly increased in patients receiving BRAF inhibitor (56, 60, 62). Additionally, freedom from ICH was also reduced in the BRAF inhibitor cohort (55). MBMs are prone to intra-tumoral hemorrhages. Up to 50% of MBM become hemorrhagic (89–91). ICH rates of 0.9 to15.2% have been associated with SRS treatment as well (78, 92). Hemorrhagic MBMs treated with SRS were found to be susceptible to local failure; hence, it may have an impact on the survival outcome (78). Surgery may be preferred in such patients, as surgery was shown to lessen the recurrence (89). BRAF inhibitors, both vemurafenib and dabrafenib, used alone have also been shown to cause intracranial hemorrhage (6%–7%) (33–35). Therefore, the combination of the two therapies may increase the odds of ICH in MBM patients. Ly et al. reported in their study that 15 of the 20 deaths attributed to CNS etiology were associated with ICH, suggesting that ICH rates may also have an impact on survival (55). Overall, these cases were managed with dose reduction, interruption, and in few cases, withdrawal.

There was no difference between the treatments in causing RN. BM patients receiving SRS treatment are at risk for RN (93, 94). As both treatment groups had received SRS, it appears that the addition of BRAF inhibitors may not be associated with an increased risk of RN in these patients. Phase 2 trials using vemurafenib and dabrafenib alone without local treatment also did not show any evidence of RN (33–35). Patel et al., however, showed an increased 1-year cumulative incidence of SRN (symptomatic RN) in patients on BRAF inhibitors. Nonetheless, in this study, an extremely low number of patients were in the BRAF inhibitor plus SRS cohort (n=15) compared to SRS alone (n=72). As the 1-year cumulative incidence of RN with SRS is 5% to 10%, it may not have enough statistical power to detect such an association (95, 96). In contrast, the 1-year cumulative incidence was significantly lower in the study by Kotecha et al. (61). Kim et al. also demonstrated a trend towards a lower incidence of RN with combined treatment than with SRS alone (BRAF inhibitor + SRS vs. SRS alone: 0% vs. 5%, p=0.20) (97). Narayan et al. also reported only one patient developing RN in 12 patients treated with vemurafenib plus SRS/WBRT (51). A similar scenario was also observed in the study by Ahmed et al., comprising 24 patients treated with vemurafenib plus SRS with only one patient reporting RN (52). In short, RN may only be associated with SRS. To confirm whether BRAF inhibitor may have a role in increasing the rate of RN, a larger study comprising such comparative groups should be undertaken.

Our study is limited by the fact that the included studies were retrospective in design. Retrospective studies are subject to confounders and tend to have selection bias, recall bias, and misclassification bias (98). Period coverage was longer for all the studies. In addition, a few studies had a small number of patients in comparative cohorts. Heterogeneity was observed in the local control outcome, and the random effects model was used for analysis.

## Future Perspective

Management of MBM is ever expanding with the addition of several BRAF and MEK targeting agents as well as the success of immune checkpoint blockade agents (5–7). SRS is becoming a predominant local therapy, and its combination with immune checkpoint blockade agents, such as ipilimumab and nivolumab have been assessed in several retrospective studies revealing an improved outcome for patients with MBM (53, 58, 59, 71–74). However, further class I evidence is needed to establish clinical guidelines. Likewise, BRAF and MEK inhibitors (alone or in combination) with SRS show promise based on the results of these retrospective studies, but further class I evidence is required (55–62). Furthermore, the efficacy of targeted agents may be further enhanced by increasing the bioavailability of these drugs in the brain. The bioavailability of several anti-cancer targeted agents, including vemurafenib and dabrafenib, have been shown to be restricted by two members of the ATP-binding cassette (ABC) family of transporters, namely P-glycoprotein (P-gp; ABCB1) and breast cancer resistance protein (BCRP; ABCG2) (37–41, 99–102). In fact, co-administration of elacridar, an ABCB1 and ABCG2 blocker, was demonstrated to improve the therapeutic efficacy of vemurafenib, especially for brain metastases located behind a functional blood-brain barrier (39). It is another area that could further enhance the effectiveness of these drugs in the brain with an improved outcome for MBM patients.

## Conclusions

Our results suggest a survival benefit for patients with MBM receiving BRAF inhibitors in conjunction with SRS as local treatment in comparison to SRS alone. Patients receiving BRAF inhibitors after SRS may have a greater survival advantage. Improvement in local control for SRS plus BRAF inhibitors may suggest that the survival surge is a result of synergism between the two treatments. BRAF inhibitors in combination with SRS may increase the risk of intracranial hemorrhage in MBM patients and warrants further investigation. Other side effects were mild in nature. Our results provide a basis for a larger randomized controlled trial to be undertaken in order to establish class I evidence.

## Data Availability Statement

The original contributions presented in the study are included in the article/**Supplementary Material**. Further inquiries can be directed to the corresponding author.

## Author Contributions

All authors listed have made a substantial, direct, and intellectual contribution to the work and approved it for publication.

## Funding

This work was supported by the Natural Science Foundation of Shenzhen (no. JCYJ20170307095828424), Shenzhen Health and Family Planning System Research Project (no. SZBC2017024), and the technical research and cultivation project for the youth of Shenzhen People’s Hospital (no. SYKYPY2019029).

## Conflict of Interest

The authors declare that the research was conducted in the absence of any commercial or financial relationships that could be construed as a potential conflict of interest.

## Supplementary Material

Supplementary Figure 1 Forest plot of meta-analysis for age difference between BRAF mutant and BRAF *wild-type*.

Supplementary Figure 2 Forest plot of meta-analysis for male sex predominance in patients with BRAF mutation and BRAF *wild-type*.

Supplementary Figure 3 Forest plot of meta-analysis for female sex predominance in patients with BRAF mutation and BRAF *wild-type*.

Supplementary Figure 4 Forest plot of meta-analysis for age difference in patients with BRAF inhibitors and Non-BRAF inhibitors.

Supplementary Figure 5 Forest plot of meta-analysis for male sex predominance in patients with BRAF inhibitors and Non-BRAF inhibitors.

Supplementary Figure 6 Forest plot of meta-analysis for female sex predominance in patients with BRAF inhibitors and Non-BRAF inhibitors.

Supplementary Figure 7 Forest plot of meta-analysis of overall survival (OS) using random effects model from diagnosis of brain metastases (BM survival) for treatment comparison (BRAF inhibitors plus SRS versus SRS alone) in the management of Melanoma brain metastatic (MBM) patients with BRAF mutant receiving BRAF inhibitors and BRAF mutant without BRAF inhibitors.

Supplementary Figure 8 Funnel plot of publication bias assessment in SRS survival analysis.

Supplementary Figure 9 Funnel plot of publication bias assessment in BM survival analysis.

Supplementary Figure 10 Funnel plot of publication bias assessment in primary diagnosis (PD) survival analysis.
