# Supplementary material for: Efficacy of BRAF Inhibitors in Combination With Stereotactic Radiosurgery for the Treatment of Melanoma Brain Metastases: A Systematic Review and Meta-Analysis
Source: Front Oncol. 2021 Feb 22;10:586029. doi: 10.3389/fonc.2020.586029 (PMC7937920; doi:10.3389/fonc.2020.586029)

**Efficacy Of BRAF Inhibitors In Combination With SRS For The Treatment Of Melanoma Brain Metastases**

**Supplementary Appendix**

This appendix has been provided by the authors to give readers additional information about their work.

**Table of Contents**

1. **Comparison of Age and gender for patients with BRAF mutant and BRAF *wild-type*………………………………………………………………………………………….…2**
   1. **FigureS1.** Forest plot of meta-analysis for age difference between BRAF mutant and BRAF *wild-type…*…………………………………………………………………………………….2
   2. **FigureS2.** Forest plot of meta-analysis for male sex predominance in patients with BRAF mutation and BRAF *wild-type…*………………………………………………………………2
   3. **FigureS3.** Forest plot of meta-analysis for female sex predominance in patients with BRAF mutation and BRAF *wild-type…*………………………………………………………………2
2. **Comparison of Age and gender for patients with BRAF inhibitors and Non-BRAF inhibitors*……………………………………………………………………………………...*3**
   1. **FigureS4.** Forest plot of meta-analysis for age difference in patients with BRAF inhibitors and Non-BRAF inhibitors*…*…………………………………………………………………...3
   2. **FigureS5.** Forest plot of meta-analysis for male sex predominance in patients with BRAF inhibitors and Non-BRAF inhibitors*…*………………………………………………………...3
   3. **FigureS6. F**orest plot of meta-analysis for female sex predominance in patients with BRAF inhibitors and Non-BRAF inhibitors*…*………………………………………………………...3
3. **Supplementary forest plots of meta-analysis of overall survival (OS)……………………4**
   1. Forest plot of meta-analysis of overall survival (OS) using random effects model from diagnosis of brain metastases (BM survival) for treatment comparison (BRAF inhibitors plus SRS versus SRS alone) in the management of Melanoma brain metastatic (MBM) patients with BRAF mutant receiving BRAF inhibitors and BRAF mutant without BRAF inhibitors……...4
4. **Funnel plot of publication bias assessment in overall survival analysis………….……….4**
   1. **FigureS7.** Funnel plot of publication bias assessment in SRS overall survival analysis……....4
   2. **FigureS8.** Funnel plot of publication bias assessment in BM overall survival analysis……...5
   3. **FigureS9**. Funnel plot of publication bias assessment in PD overall survival analysis…….....5

**Supplementary Appendix 1.**

**Comparison of Age and gender for patients with BRAF mutant and BRAF *wild-type.***

1.1 **FigureS1.** Forest plot of meta-analysis for age difference between BRAF mutant and BRAF *wild-type.*


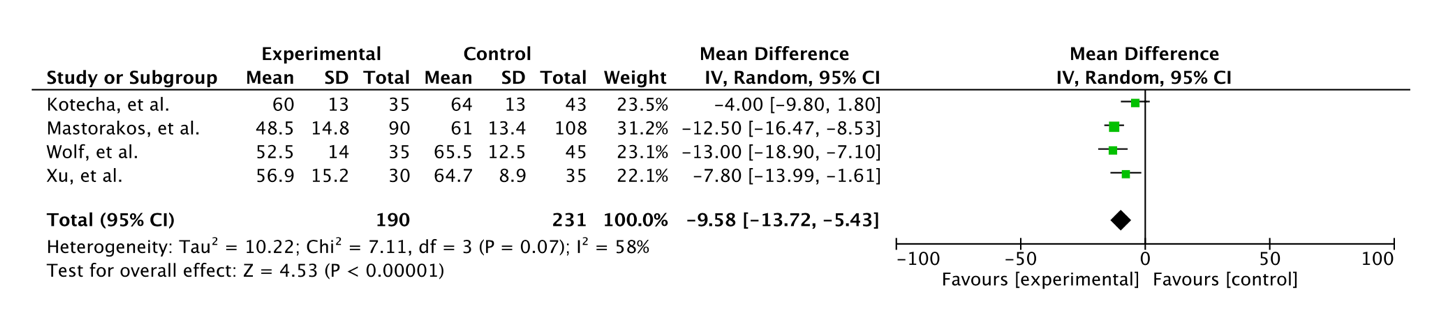


1.2 **FigureS2.** Forest plot of meta-analysis for male predominance in patients with BRAF mutation and BRAF *wild-type.*


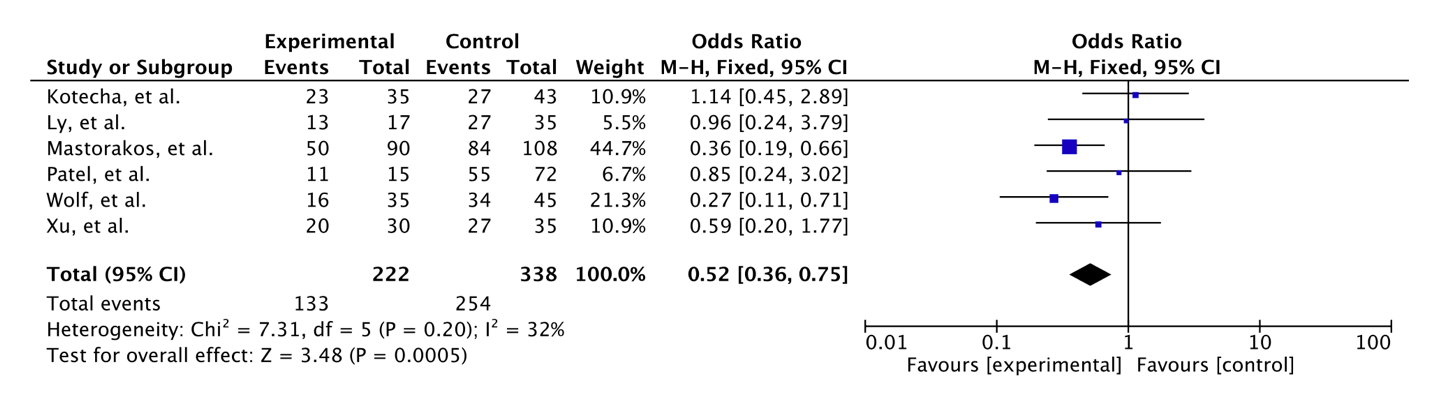


1.3 **FigureS3.** Forest plot of meta-analysis for female predominance in patients with BRAF mutation and BRAF *wild-type.*


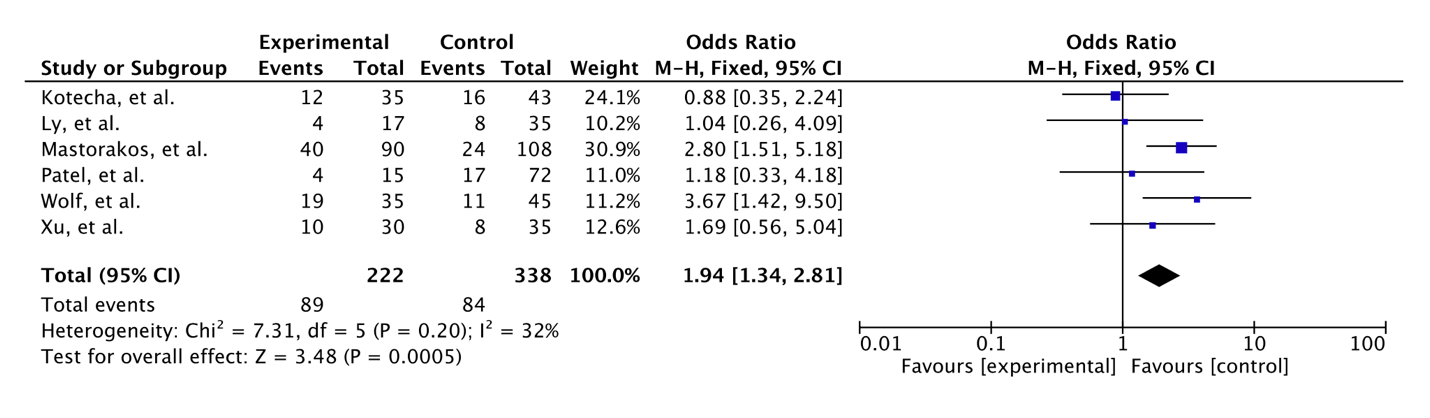


**Supplementary Appendix 2.**

**Comparison of Age and gender for patients with BRAF inhibitors and Non-BRAF inhibitors*.***

2.1 **FigureS4.** Forest plot of meta-analysis for age difference in patients with BRAF inhibitors and Non-BRAF inhibitors*.*


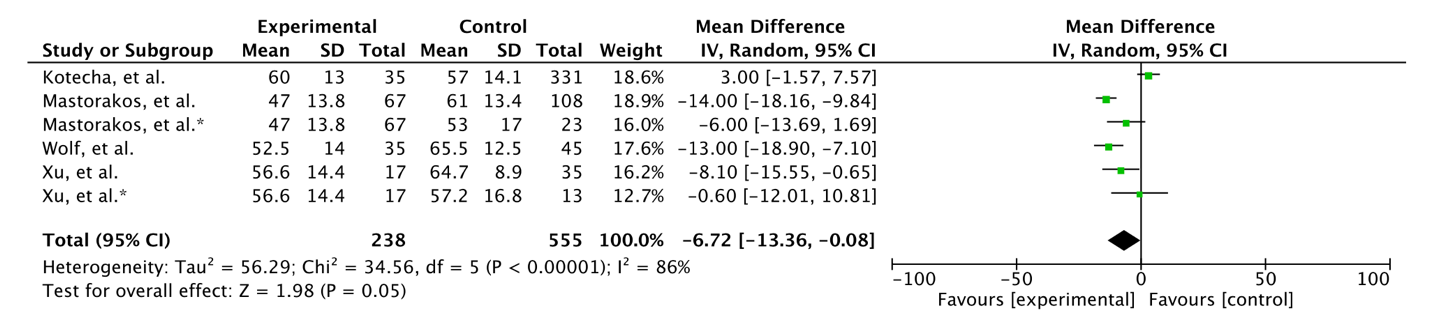


2.2 **FigureS5.** Forest plot of meta-analysis for male predominance in patients with BRAF inhibitors and Non-BRAF inhibitors*.*


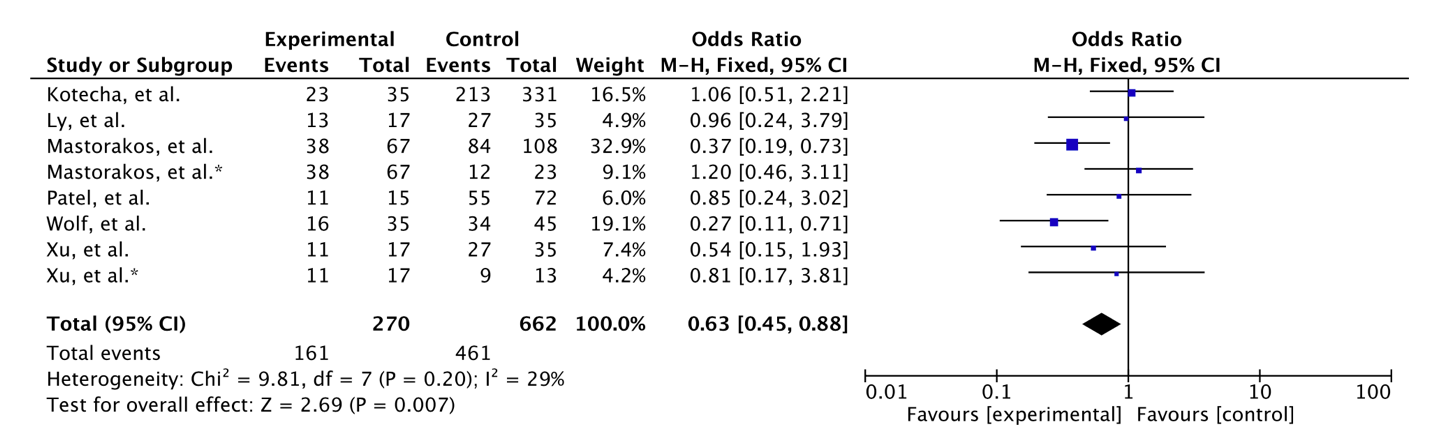


2.3 **FigureS6. F**orest plot of meta-analysis for female predominance in patients with BRAF inhibitors and Non-BRAF inhibitors*.*


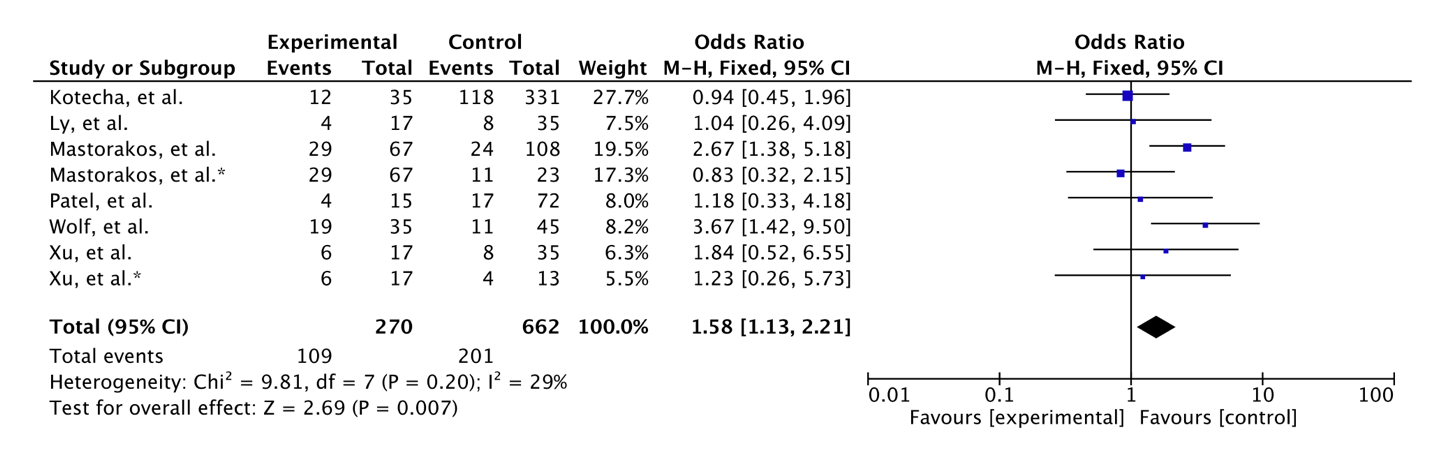


**Supplementary Appendix 3.**

**Supplementary forest plots of meta-analysis of overall survival**

3.1 **FigureS7.** Forest plot of meta-analysis of overall survival (OS) using random effects model from diagnosis of brain metastases (BM survival) for treatment comparison (BRAF inhibitors plus SRS versus SRS alone) in the management of Melanoma brain metastatic (MBM) patients with BRAF mutant receiving BRAF inhibitors and BRAF mutant without BRAF inhibitors


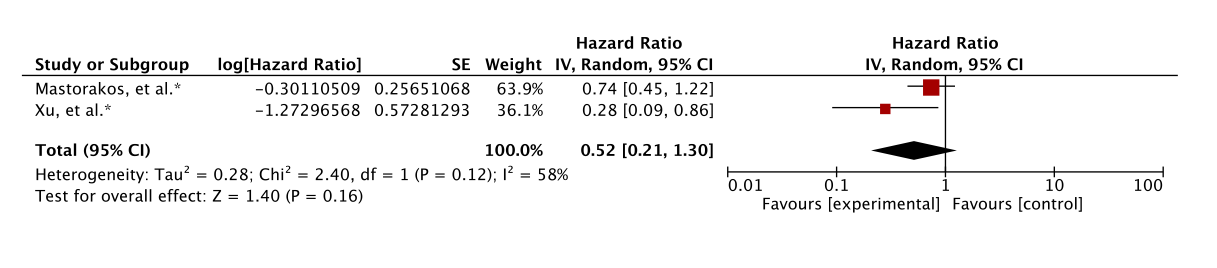


**Supplementary Appendix 4.**

**Funnel plot of publication bias assessment in overall survival analysis**

4.1 **FigureS8.** Funnel plot of publication bias assessment in SRS overall survival analysis. All studies are within the 95% confidence interval.


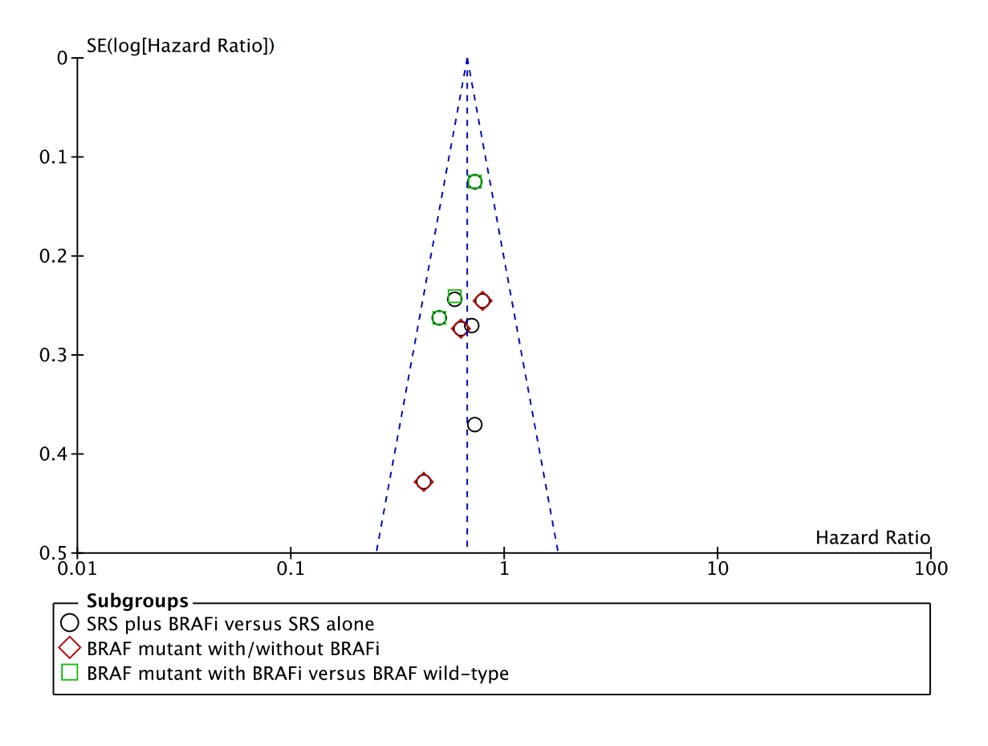


**FigureS9.** Funnel plot of publication bias assessment in BM overall survival analysis. All studies are within the 95% confidence interval.


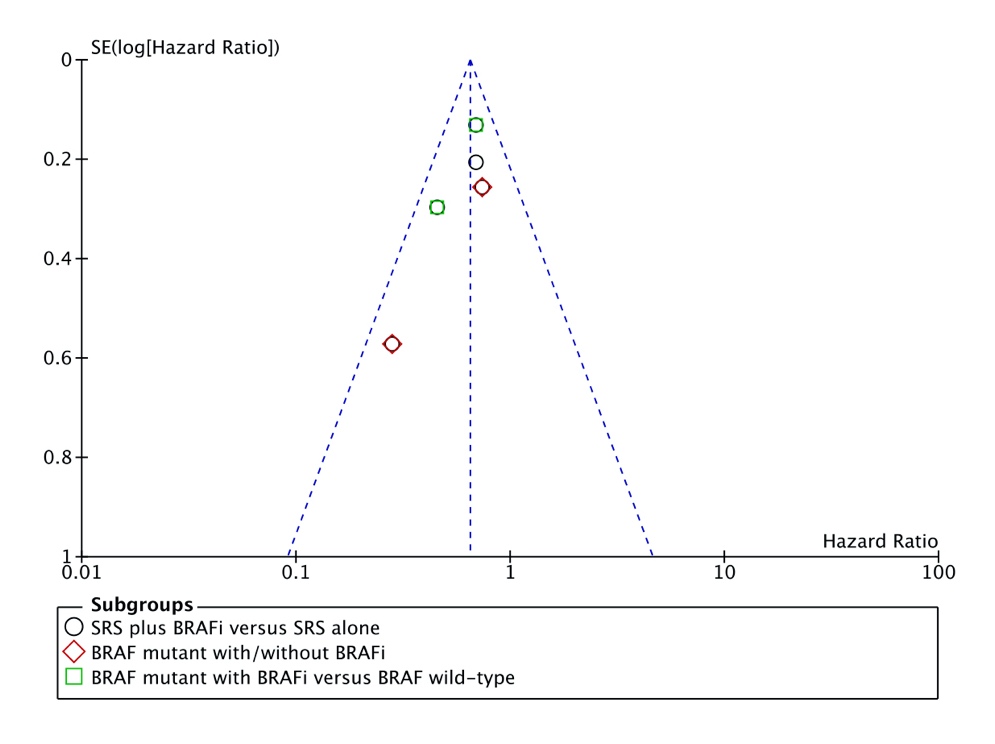


**FigureS8.** Funnel plot of publication bias assessment in BM overall survival analysis. All studies are within the 95% confidence interval.


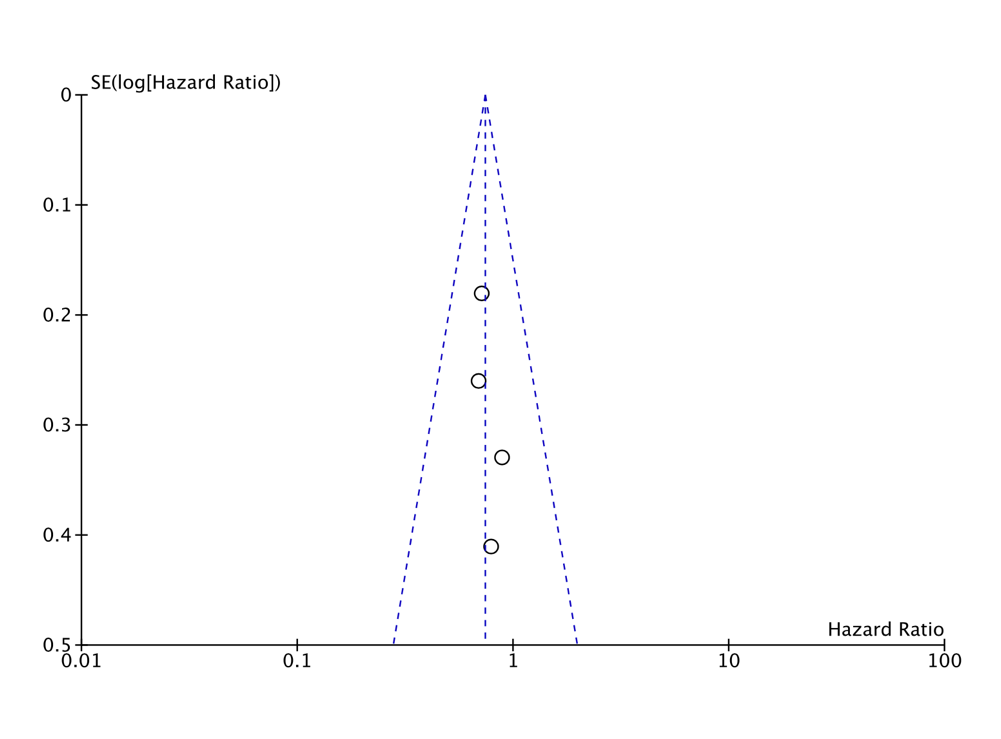

Supplement: Supplementary Figure 1 — Forest plot of meta-analysis for age difference between BRAF mutant and BRAF wild-type. [file DataSheet_1.docx]
